# Neglected tropical diseases in the People’s Republic of China: progress towards elimination

**DOI:** 10.1186/s40249-019-0599-4

**Published:** 2019-10-02

**Authors:** Men-Bao Qian, Jin Chen, Robert Bergquist, Zhong-Jie Li, Shi-Zhu Li, Ning Xiao, Jürg Utzinger, Xiao-Nong Zhou

**Affiliations:** 1 National Institute of Parasitic Diseases, Chinese Center for Disease Control and Prevention, Chinese Center for Tropical Diseases Research, Key Laboratory of Parasite and Vector Biology, Ministry of Health, National Center for International Research on Tropical Diseases, Ministry of Science and Technology, WHO Collaborating Center for Tropical Diseases, Shanghai, People’s Republic of China; 2 Ingerod, Brastad, Sweden; 3 Key Laboratory of Surveillance and Early-warning on Infectious Disease, Chinese Center for Disease Control and Prevention, Beijing, People’s Republic of China; 4 Swiss Tropical and Public Health Institute, Basel, Switzerland; 5 University of Basel, Basel, Switzerland

**Keywords:** Control, Elimination, People's Republic of China, Neglected tropical diseases

## Abstract

Since the founding of the People’s Republic of China in 1949, considerable progress has been made in the control and elimination of the country’s initial set of 11 neglected tropical diseases. Indeed, elimination as a public health problem has been declared for lymphatic filariasis in 2007 and for trachoma in 2015. The remaining numbers of people affected by soil-transmitted helminth infection, clonorchiasis, taeniasis, and echinococcosis in 2015 were 29.1 million, 6.0 million, 366 200, and 166 100, respectively. In 2017, after more than 60 years of uninterrupted, multifaceted schistosomiasis control, has seen the number of cases dwindling from more than 10 million to 37 600. Meanwhile, about 6000 dengue cases are reported, while the incidence of leishmaniasis, leprosy, and rabies are down at 600 or fewer per year. Sustained social and economic development, going hand-in-hand with improvement of water, sanitation, and hygiene provide the foundation for continued progress, while rigorous surveillance and specific public health responses will consolidate achievements and shape the elimination agenda. Targets for poverty elimination and strategic plans and intervention packages post-2020 are important opportunities for further control and elimination, when remaining challenges call for sustainable efforts.

## Multilingual abstracts

Please see Additional file 1 for translations of the abstract into the five official working languages of the United Nations.

## Background

The term “neglected tropical diseases” (NTDs in short) was coined some 15 years ago, referring to a diverse group of diseases that are intimately linked to poverty and primarily occur in tropical and subtropical countries, affecting marginalized communities in rural and deprived urban settings [1, 2]. In the meantime, considerable progress has been made in the control of NTDs with some of them having been targeted for elimination [3]. Yet, the NTDs remain a public health problem and drain the social and economic development in many parts of the world [4].

In its first report issued in 2010, the World Health Organization (WHO) listed 17 NTDs [5]. In the meantime, the list has been expanded to 20 NTDs, which are caused by a diverse set of agents, such as bacteria, parasites, and virus, in addition to snakebites [6]. Over a billion people are affected by one or several NTDs, primarily in low- and middle-income countries (LMICs) [4, 6]. Most of the NTDs are tracked by the Global Burden of Diseases (GBD) Study, and hence, their global burden, as expressed in disability-adjusted life years (DALYs), is updated annually. Taken together, the global burden of the NTDs in 2017 was estimated at 17 million DALYs [7] .

In 2012, WHO released a roadmap for the control and elimination of the NTDs, which included specific milestones for achieving set goals [8]. Inspired by this roadmap, the London Declaration was released in the same year and several institutions, foundations, philanthropic organisations, and pharmaceutical companies pledged support to foster research and development on NTDs, and to assist WHO to work with endemic countries, non-governmental organisations (NGOs), and influential politicians towards control and elimination of 10 NTDs (i.e. eradication of Guinea worm; elimination as a public health problem of lymphatic filariasis, leprosy, human African trypanosomiasis, and blinding trachoma; and control of schistosomiasis, soil-transmitted helminth infection, Chagas disease, visceral leishmaniasis, and onchocerciasis by 2020) [9]. Reports note that, despite existing challenges, the potential to accelerate progress towards Universal Health Coverage (UHC) remains in place with endorsement by WHO [3, 10, 11]. Integration of activities and interventions into broader health systems is being promoted, and the agenda has been advanced to 2030 [3].

Historically, many of the diseases discussed here have dominated the public health agenda in the People’s Republic of China. Eleven of the NTDs currently listed by WHO have been and several continue to be endemic, thus negatively impacting on people’s health and wellbeing (Table 1). Yet, owing to sustained social and economic development, going hand-in-hand with massive control efforts, the influence of these NTDs have been significantly reduced with some of them already eliminated [12]. Indeed, the elimination of lymphatic filariasis as a public health problem, achieved by the People’s Republic of China as the first country in the world and verified by WHO in 2007, is a major success story [13]. In 2015, the elimination of trachoma as a public health problem followed suit [14], while substantial progress has been made with respect to the remaining nine NTDs. The government is committed to further strengthen control activities and move towards elimination of most of the remaining NTDs by or beyond 2020.

**Table 1:** Progress in the control and elimination of NTDs in the People’s Republic of China

Infection	Estimated number of infections/new infections (year)	Current geographical distribution	Coverage of active surveillance	Coverage of passive surveillance
Bacterial				
Leprosy	634 new cases (2017)	18 provinces, mainly in western and southern areas	No	National level
Trachoma	Eliminated as public health problem (2015)	–	No	No
Viral				
Dengue	5893 new cases (2017)	27 provinces, mainly in 3 southern provinces	5 provinces	National level
Rabies	516 new cases (2017)	27 provinces, mainly in eastern, central, and southwestern areas	6 provinces	National level
Protozoal				
Leishmaniasis	182 new cases (2017)	10 provinces, mainly in 3 western indigenous provinces	No	National level
Helminth				
Echinococcosis	166 098 (2012–2016)	9 western provinces	All 9 endemic provinces	National level
Food-borne trematodiasis				
Clonorchiasis	6.0 million (2014–2015)	18 provinces, mainly in southeastern and northeastern areas	30 provinces	No
Lymphatic filariasis	Eliminated as a public health problem (2007)	–	No	National level
Schistosomiasis	37 601 (2017)	12 provinces along the Yangtze River	13 provinces	National level
Soil-transmitted helminth infection	29.1 million (2014–2015)	31 provinces, mainly in the western and southern parts	30 provinces	No
Ascariasis	8.8 million (2014–2015)	31 provinces, mainly in western areas		
Trichuriasis	6.6 million (2014–2015)	28 provinces, mainly in western areas		
Hookworm infection	17.0 million (2014–2015)	19 provinces, mainly in the western and southern parts		
Taeniasis/cysticercosis				
Taeniasis	36 6247^a^ (2014–2015)	12 provinces, mainly in western areas	5 provinces	No
Cysticercosis	No clear data available	Mainly in western areas	No	No

*NTDs* neglected tropical diseases

^a^ Most estimated to be *T. saginata*

Clearly, experiences and lessons from the People’s Republic of China are relevant for the control and elimination of NTDs elsewhere [15]. To that end, we review here the progress and milestones of the control and elimination of NTDs since the foundation of the People’s Republic of China exactly 70 years ago, at the onset of the Belt and Road Initiative. The accomplishments with reference to the achievement of targets expected by 2020 are summarised, the remaining challenges highlighted, and opportunities for sustained control and elimination of the NTDs emphasised.

## Progress in the control and elimination of NTDs in the People’s Republic of China

### Bacterial infections

#### Leprosy

**Fig. 1:**
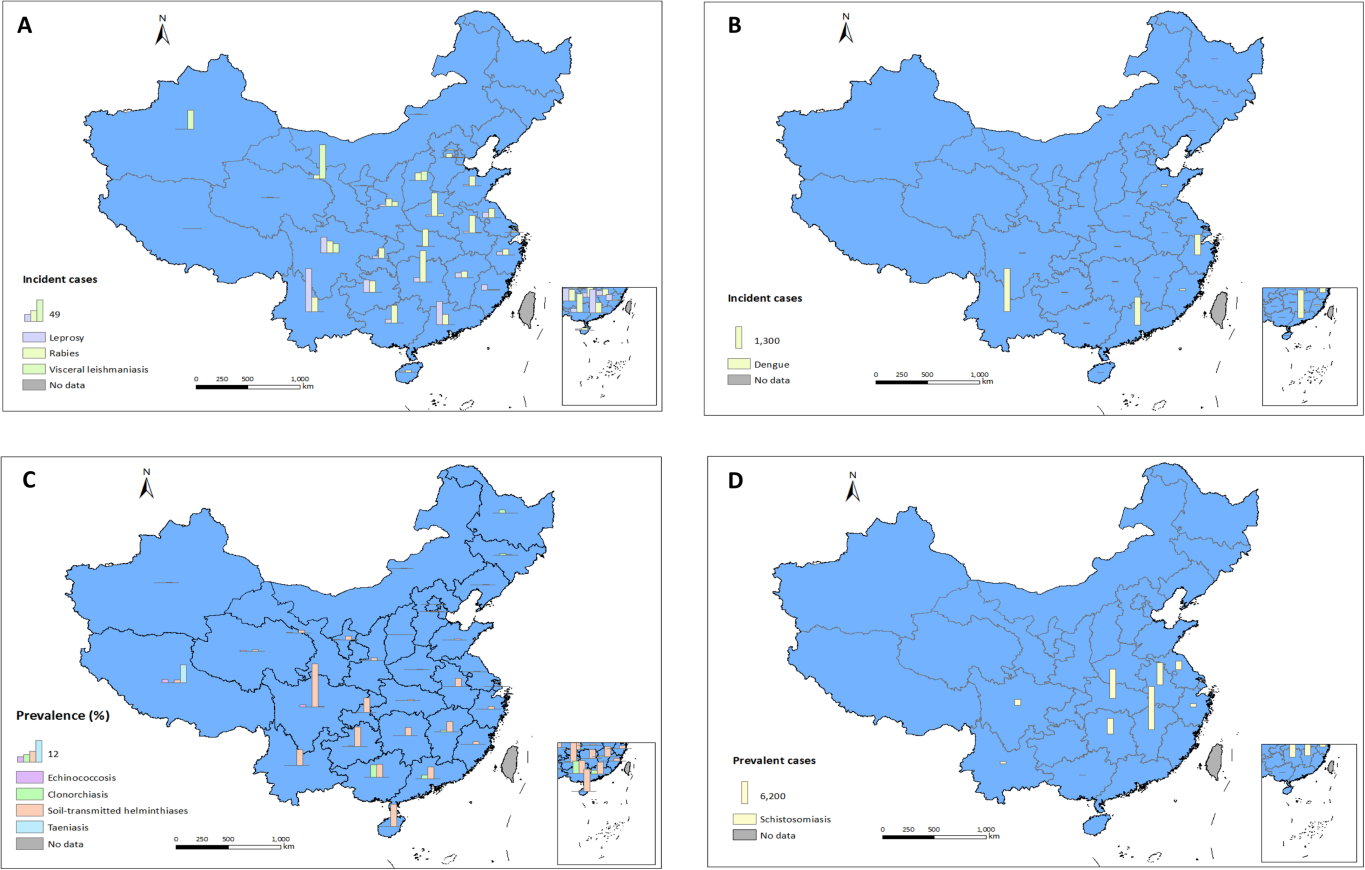
Endemicity of NTDs, stratified by province, in the People’s Republic of China. **a** Reported cases of leprosy, rabies, and visceral leishmaniasis from passive surveillance (in 2017); **b** reported cases of dengue from passive surveillance (in 2017); **c** prevalence of echinococcosis, clonorchiasis, soil-transmitted helminth infection, and taeniasis from national surveys (between 2012 and 2016); and **d** estimated cases of schistosomiasis (in 2017). NTDs: neglected tropical diseases

**Fig. 2:**
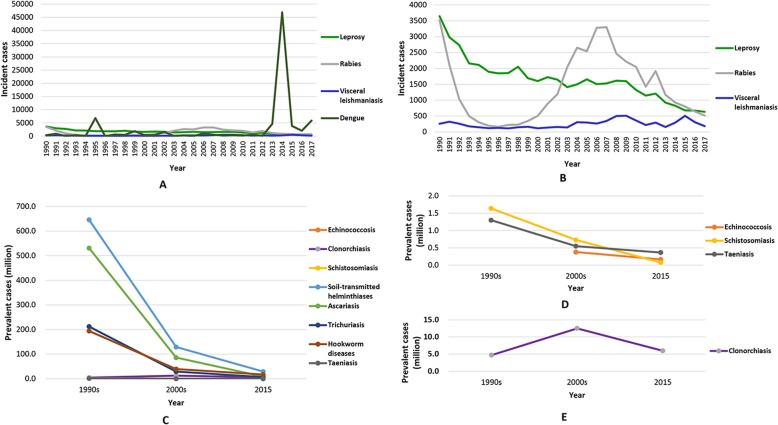
Change trends of NTDs in the People’s Republic of China. **a** Reported cases of leprosy, rabies, visceral leishmaniasis, and dengue from passive surveillance (1990–2017); **b** reported cases of leprosy, rabies, and visceral leishmaniasis from passive surveillance (1990–2017); **c** estimated cases of echinococcosis, clonorchiasis, schistosomiasis, soil-transmitted helminth infection. and taeniasis from national surveys (1990s–2015); **d** estimated cases of echinococcosis, schistosomiasis, and taeniasis from national surveys (1990s–2015); and **e** estimated cases of clonorchiasis from national surveys (1990s–2015). NTDs: Neglected tropical diseases

About 500 000 leprosy cases were detected in the People’s Republic of China between 1949 and 2017, mainly in the south-western and south-eastern parts of the country (Table 1 and Fig. 1a) [16–20]. The annual detection rate of new cases exceeded 1 per 100 000 between 1954 and 1976, with a peak of 5.56 per 100 000 (34 878 cases in total) in 1958 [16]. At the initial control stage, from the 1950s to 1980, an approach consisting of detection, separation, and treatment was employed to control the infection [21]. Incidence started to decline already around 1970 and continued to drop. A second phase began in 1981 with the stated aim to eliminate leprosy, targeting a prevalence level of < 1 per 100 000 at county level through early case detection and multidrug therapy [21]. In 2017, an all-time low of 634 new cases were reported, corresponding to a incidence of 0.05 per 100 000 (Fig. 2a and b) [20]. Meanwhile, the countrywide prevalence reached 0.19 per 100 000 (2697 cases), with most of the cases (*n* = 1643) concentrated in five province in the southern part of the People’s Republic of China [20].

In 2011, the “Programme on the elimination of leprosy in China 2011–2020” was launched, promoting professional training, early detection, and regulatory treatment, combined with setting-specific information, education, and communication (IEC) [22]. Two specific targets were included: (i) halving the total number of cases in 2010 by 2020 (i.e. 3300) and reducing the prevalence below 1 per 100 000 in 98% counties [22]. The first target was already achieved in 2015 (i.e. 3230 cases) [19].

#### Trachoma

Before 1949, it was estimated that the prevalence of trachoma in the People’s Republic of China was about 50%, with 25–37% of all blindness in the country caused by this bacterial infection [23]. From 1956 onwards, trachoma control was prioritised by inclusion in the National Programme of Agricultural Development (1956–1967) [24], which encouraged hygienic measures, such as regularly cleaning hands and faces with running water and use of individual towels, among other public health measures [23]. In 1990, the control of trachoma became part of school health regulations [25]. The “SAFE” strategy recommended by WHO was also gradually adopted, consisting of surgery for advanced disease (S), antibiotics to clear *Chlamydia trachomatis* infection (A), facial cleanliness (F), and environmental improvement to reduce transmission (E). According to two national disability surveys, the prevalence of blindness caused by trachoma decreased to 51.5 per 100 000 in 1987 and to 17.6 per 100 000 in 2006, at the same time as the proportion of blindness attributed to trachoma decreased to 10.1% and to 0.9%, respectively [26].

From September 2012 onwards, the goal “Eliminating blinding trachoma in China before 2016” was promoted [23]. Within two years, 8163 children under the age of nine years in 130 schools, and 87 924 355 residents aged 15 years and above in 55 679 villages were surveyed in 16 provinces with a previously high trachoma incidence [14]. The prevalence of active trachoma and trichiasis (abnormally positioned eyelashes) decreased to 0.2% and 0.002%, respectively, thereby reaching the targets set by WHO for elimination of trachoma as a public health problem [14].

### Viral infections

#### Dengue

Although dengue was endemic in the People’s Republic of China before 1949, it disappeared for nearly 30 years until an outbreak occurred in 1978 in Guangdong province in the southern part of the country, which affected 22 122 people [27]. In 1980, another, even much larger outbreak hit Guangdong, affecting 452 674 individuals [27]. Overall, more than 660 000 people were infected in Guangdong between 1978 and 1991, causing 493 deaths [27]. This led to dengue being included among the national notifiable diseases from 1989 onwards. During 1990–2017, a total of 80 583 cases (including 13 deaths) were reported through the health system (Fig. 2a) [20, 28, 29]. Although the situation improved in the 1980s, indigenous cases expanded from southern coastal tropical or subtropical regions to neighbouring northern and western regions, reaching as far as the central part of the People’s Republic of China (Table 1 and Fig. 1b) [28]. Additionally, imported cases (both from domestic endemic areas and overseas) occurred with a nationwide distribution [28]. Another large outbreak hit Guangdong province in 2014, involving 45 224 cases and six deaths [30].

It is conceivable that the pre-1978 disappearance of dengue is attributable to effective mosquito control efforts facilitated by Patriotic Health Campaigns [31]. Population mobilization, imported cases, and vector multiplication caused subsequent outbreaks. Surveillance, timely management of cases, and control of vectors remain the key measures against dengue [32].

#### Rabies

Rabies is primarily caused by dog bites (95%); yet, cat bites also contribute (4%) [33]. From 1960 to 2017, a total of 122 874 cases of rabies were reported in the People’s Republic of China [20, 29, 34]. Between 1979 and 1989, over 4000 cases were reported every year, with a peak in 1981 (7037 cases; 0.71 per 100 000) [34]. The disease decreased gradually and reached its lowest level in 1996 (159 cases; 0.01 per 100 000) [34]. However, it increased once again reaching a peak in 2007 (3300 cases; 0.25 per 100 000) [33, 34] after which a gradual decrease to 516 cases in 2017 was noted (Table 1, Fig. 2a and b) [20, 29], with most cases found in the eastern and southern regions (Fig. 1a). In spite of the recent overall decrease, rabies expanded slightly in the western and northern areas over the past 10 years. Rural areas are most severely afflicted [34].

The fluctuating number of infections is explained by multiple factors. For example, the increasing numbers of household dogs without immunization and the transportation of dogs might explain the two peaks of rabies noted in the 1980s and the 2000s. Investment in training of health professionals and an increasing access to post-exposure prophylaxis in rural areas led to the recent, sustainable decrease [33]. The central government’s mid- and long-term control strategy for epidemic diseases in animals (2012–2020) requires national control of rabies by 2020, mainly promoted through dog immunization [35]. In 2017, the Ministry of Agriculture issued a national control plan (2017–2020), aiming at strengthening rabies control by targeting coverage of 90% immunization of registered dogs in all counties by 2020 [36].

### Protozoa infections

#### Leishmaniasis

Between 1951 and 1972, visceral leishmaniasis was endemic in 16 provinces in the northern part of the People’s Republic of China and along the Yangtze River with the main endemic areas located in the plain region of the central and eastern parts, the North China Plain and the Central Shaanxi Plain [37]. A conservative estimate in 1951 indicated that 530 000 people were infected [37]. The disease was given a high priority and included in the National Programme of Agricultural Development 1956–1967 [24]. Considerable efforts were made to control the disease, including the establishment of professional leishmaniasis control groups tasked with the detection and treatment of patients and insecticide spraying to eliminate the sand fly vector [37]. No new cases were detected in the previously endemic plains since 1983, but endemicity persisted in the hilly and desert areas of six western provinces (Fig. 1a) [37]. Additionally, imported cases were reported from other provinces. For example, 3994 cases were reported from 27 provinces in the period 2005–2015, with a percentage of 95.3% in Xinjiang, Sichuan, and Gansu provinces, which remain highly endemic, especially Xinjiang where two outbreaks were reported in the same county (Jiashi) in 2008 and 2015 [38, 39]. Nationally, however, the prevalence is low and diminishing: 305 and 182 cases were reported in 2016 and 2017, respectively (Table 1, Fig. 2a and b) [20, 29].

The less serious form of the disease, cutaneous leishmaniasis, has also been reported in the People’s Republic of China, but is confined to a single isolated site; namely, Karamay county in Xinjiang. The detection rate was 1.6% (36 per 2260) in 1992, 1.0% (14 per 1416) in 1993, and 1.6% (24 per 1510) in 1994 in Karamay, respectively [40]. We are not aware of more recent data.

Incidentally, the “National control plan on echinococcosis and other important parasitic diseases” for the period 2016–2020 is required to also attempt decreasing the burden of visceral leishmaniasis through detection and treatment of cases, provision of long-lasting insecticidal nets, surveillance, and integrated vector management [41].

### Helminthiases

#### Echinococcosis

Echinococcosis, caused by either *Echinococcus granulosus* or *E. multilocularis*, is endemic in the western part of the People’s Republic of China (Table 1 and Fig. 1c). According to a survey carried out in 2001–2004, there were an estimated 380 000 cases in eight provinces, with an average prevalence of 1.1% (Fig. 2c and d) [42]. This led to the initiation of the national echinococcosis control programme in 2005 with support from the central government. By 2014, 254 highly endemic counties were included in the programme [43]. A total of 666 million Chinese Yuan (about US$ 100 million) was allocated for the control of echinococcosis between 2010 and 2014 [43], and the number of ultrasound examinations increased from 1.52 million to 2.13 million between 2011 and 2014. An updated survey covering the years 2012–2016 showed that the total number of estimated cases had decreased to 166 098, with a prevalence of 0.3% in nine western provinces [44, 45]. However, 368 counties still remain endemic with cystic echinococcosis, out of which 115 are co-endemic with alveolar echinococcosis [44, 45].

In the latest national control plan for the period 2016–2020, control of echinococcosis has been further intensified, integrating the control of the transmission source by deworming of dogs, immunization of livestock, and screening and management of patients [41]. The goal set for 2020 is to reach over 70% endemic counties reducing the prevalence to less than 1% in humans and less than 5% in domestic dogs.

#### Food-borne trematodiasis

##### Clonorchiasis

Clonorchiasis, caused by consumption of raw or undercooked freshwater fish, is the most important food-borne trematode infection in the People’s Republic of China [46]. The first national survey conducted in 1988–1992 estimated a national prevalence of 0.31%, which increased to 0.58% in the second national survey done in 2001–2004 [47]. However, an additional survey carried out in the most affected regions at the same time, found a considerably higher prevalence of 2.4%, and hence, it was estimated that 12.5 million people were infected with the liver fluke *Clonorchis sinensis* (Fig. 2c and e) [47]. These increasing trends are felt to be attributable to economic development, which spurred rapid expansion of aquaculture, and hence, freshwater fish has become a diet that can be afforded by many more people [48]. In recent years, control activities were adopted in the endemic areas, consisting of chemotherapy, IEC, and improved sanitation [49, 50], which might explain the reduction in the prevalence to 0.47% corresponding to 6.0 million people according to the latest national survey in 2014–2015 [51]. The most afflicted areas, with prevalence rates over 1%, are two provinces in the south-eastern part of the People’s Republic of China (Guangdong and Guangxi) and two in the north-eastern part (Heilongjiang and Jilin) (Table 1 and Fig. 1c) [51]. Compared to 2014–2015, the national control plan for 2016–2020 aims to decrease the prevalence in all major endemic provinces by 30% in 2020 through implementation of an integrating control strategy, consisting of chemotherapy, IEC, and improved sanitation [41].

#### Paragonimiasis

Paragonimiasis, caused by the lung fluke *Paragonimus westermani*, is a common food-borne trematode infection in the People’s Republic of China [52]. *P. skrjabini* is another important species that mainly occurs in the southern parts of the People’s Republic of China. It can cause trematode larva migrans, which helps for species-specific diagnosis [52]. Transmission of paragonimiasis occurs by the traditional habit of consuming raw, wine-soaked crab meat, so called “drunken crabs” in the southern part of the country; raw crab meat, raw crab-sauce, or crab-jam in the south-western part of the country; and raw crayfish and crayfish-curd in the north-eastern part of the country [53]. A large serological survey in eight endemic provinces between 2001 and 2004 showed a prevalence of 1.7% [42]. However, the 2014–2015 national survey based on faecal examination showed a crude prevalence of only 0.005% [51]. It is conceivable that the serological survey considerably overestimated the prevalence due to the low specificity of this approach, particularly in view of co-endemicity with other helminth infections. With regard to faecal examination, it is likely to have underestimated the true infection status due to low sensitivity. Thus, the establishment of a national endemic map for paragonimiasis constitutes a pressing public health priority. Screening and treatment of cases, combined with IEC, constitute the current mainstay of control measures [53].

#### Fascioliasis

Fascioliasis is endemic in the northern, central, and southern parts of the People’s Republic of China with both *Fasciola hepatica* and *F. gigantica* reported [54, 55]. Watercress and *Houttuynia cordata*, which cause the infection in humans when ingested raw, are widely consumed in the endemic areas. A prevalence of 0.011% was reported in the national survey conducted in 1988–1992, with cases found in eight provinces [56], while it decreased to 0.0007% in the national survey done in 2014–2015 [51]. Although the overall prevalence is very low, several outbreaks have been reported recently [55, 57]. The low awareness of this infection in the public usually leads to severe delays in the diagnosis and subsequent management of the disease. Currently, the surveillance of outbreaks and specific public health responses is applied in some major endemic areas (e.g. Yunnan province in the south-western part of the People’s Republic of China).

#### Lymphatic filariasis

Historically, lymphatic filariasis was endemic in the central and south-eastern parts of the People’s Republic of China, either caused by *Wuchereria bancrofti* or *Brugia malayi* [13, 58]. Lymphatic filariasis was endemic in 864 counties in 16 provinces covering a population of approximately 330 million [13, 58]. In the 1950s, the number of patients was estimated at 31.0 million, among which 25.6 million had microfilaraemia and 5.4 million were classified as clinical cases [58, 59]. Owing to the inclusion in the National Programme of Agricultural Development 1956–1967, massive control activities were implemented against lymphatic filariasis [59]. A strategy to eliminate the transmission source through large-scale chemotherapy was employed, based on the dearth of animal reservoirs [60]. During the period 1956–1994, there were 707.4 million blood examinations with 23.4 million of the blood samples tested found to have microfilaraemia [58]. Overall, there were 260.0 million contact points for chemotherapy (including 33.9 million by individual treatment, 31.6 million by mass drug administration, and 194.5 million by fortified salt) [58]. In 1994, all 864 counties reached a microfilaria prevalence below 1% at the unit of the village, which is considered the threshold for breaking transmission [58].

Since the early 1980s, surveillance was gradually established at the provincial level when province-wide effective control of lymphatic filariasis had been achieved [13]. Elimination was first announced in Guangxi in 1995 and the last province to do so was Anhui in 2006 [13]. In the same year, the Chinese government submitted its dossier for lymphatic filariasis elimination to WHO. In 2007, WHO declared that the People’s Republic of China had succeeded to eliminate lymphatic filariasis as a public health problem [13]; the first country in the world.

#### Schistosomiasis

Schistosomiasis in the People’s Republic of China is exclusively caused by infection with the species *Schistosoma japonicum*. In the 1950s, schistosomiasis was endemic in 12 southern provinces, primarily along the Yangtze River and it was estimated that 11.6 million people were infected [61]. Over the past 60 years, schistosomiasis has been the focus of concerted public health efforts steered by the central government [62]. Four stages of the national schistosomiasis control programme can be distinguished: (i) the preparation stage (1950–1955); (ii) the mass campaign stage focusing on snail control (1956–1985); (iii) the morbidity control stage emphasising large scale administration of praziquantel, boosted by international cooperation (1986–2003); and (iv) the current integrated strategy to block infection transmission (since 2004) [63]. By 1989, the prevalence and the number of cases infected with *S. japonicum* decreased to 10.2% and 1.64 million, respectively (Fig. 2c and d). The respective estimates for 1995 were 4.9% and 870 000, while, in 2004, the prevalence and number of cases further declined to 2.5% and 730 000, respectively [61, 64]. Five provinces achieved transmission interruption; Shanghai and Guangdong (both in 1985), Fujian (in 1987), Guangxi (in 1988), and Zhejiang (in 1995) [65]. Especially, the successful integrated strategy to control transmission source adopted in 2004 led to a stage that can be considered pre-elimination. In 2017, it was estimated that only 37 601 cases remained in the country (Table 1 and Fig. 1d) [66]. In addition to the five provinces where elimination was declared, one reached interruption of transmission, while the remaining six reached transmission control in 2017 [66]. Another four provinces are targeted to achieve transmission interruption by 2020 by integrating control of transmission sources and environmental management [67]. Additionally, surveillance is prioritised in the areas reaching the stage of transmission interruption and elimination. The ambitious target set by the central government is to achieve complete elimination of schistosomiasis in the People’s Republic of China by 2030 [68].

#### Soil-transmitted helminth infection

Soil-transmitted helminth infections occur throughout the People’s Republic of China; yet, the most severely affected provinces are located in the southern parts of the country, explained by climatic and ecological features. Over the past 30 years, a significant decrease of soil-transmitted helminth infection has been documented unequivocally according to three national surveys. The first survey, taking place between 1988 and 1992, showed a prevalence of 47.0% for *Ascaris lumbricoides* infection, 18.8% for *Trichuris trichiura* infection, and 17.2% for hookworm infection and overall infection of any soil-transmitted helminth infection of 53.6% [56, 69]. The corresponding numbers of people infected were estimated at 531 million, 212 million, 194 million, and 646 million, respectively (Fig. 2c) [56, 69]. In the second national survey conducted between 2001 and 2004, the respective prevalence rates dropped to 12.7, 4.6, 6.1, and 19.6%, with estimated numbers of people of 85.9 million, 29.1 million, 39.3 million, and 129.0 million, respectively [42]. In the third survey carried out in 2014–2015, the prevalence had further declined to 1.4, 1.0, 2.6, and 4.5% with the numbers of infected people estimated at 8.8 million, 6.6 million, 17.0 million, and 29.1 million, respectively [51]. At present, soil-transmitted helminth infections are concentrated in the south-western parts of the People’s Republic of China (Table 1 and Fig. 1c) [51].

The significant decrease of soil-transmitted helminth infection is primarily attributable to social and economic development that spurred improved access to clean water, sanitation, and hygiene (WASH), coupled with other far-reaching control activities. Since the early 1990s, massive control activities were applied in schools, including IEC, WASH, and preventive chemotherapy [70]. Integrated community pilots, based on a multifaceted control strategy similar to that implemented in the schools, were employed in eight highly endemic counties since 2006 [71]. The strategy proved successful in terms of prevalence reduction, and hence, was scaled up over the past several years [71]. According to the national programme, the prevalence of soil-transmitted helminth infection in the remaining high-risk areas should decrease by another 20% by 2020 compared to 2015 [41].

#### Taeniasis/cysticercosis

Cysticercosis is primarily caused by larval cysts of the tapeworm *Taenia solium*. Historically, the disease was highly endemic in the People’s Republic of China, especially in the northern parts. Consequently, the Office for Controlling and Eliminating Taeniasis and Cysticercosis was established in most of the endemic provinces during the 1970s, 1980s, and early 1990s [72]. The integrated control strategy consisted of four main interventions: (i) deworming people with taeniasis; (ii) inspection of pork meat; (iii) management of human faeces and pigs; and (iv) treatment and disposal of infected pigs [72]. Rigorous implementation of this control strategy resulted in a significant decrease in many provinces. However, there is still no national endemic map available for cysticercosis.

In 2001–2004, a national serological survey was implemented, which revealed a crude positive rate of 0.58% (553/96008) [42]. Due to the low test specificity, it is conceivable that the prevalence is considerably overestimated. In view of relatively scarce data available from hospitals, cysticercosis is concentrated in the provinces of Yunnan and Sichuan in the south-western part of the country, which is explained by the deeply rooted cultural habit of raw pork consumption [73].

The national survey on intestinal parasitic diseases based on faecal examination has clarified the situation with regard to taeniasis in the People’s Republic of China. An overall prevalence of 0.01% was found in 1988–1992, corresponding to approximately 1.3 million cases (Fig. 2c and d) [56, 74]*.* The 2001–2004 survey estimated a considerably lower number of 550 000 cases, which further decreased to 366 200 cases in 2014–2015 [42, 51]. Of note, besides *T. solium*, another two species (i.e. *T. saginata* and *T. asiatica*) are co-endemic in the People’s Republic of China [73]. In the 1988–1992 survey, using species differentiation, only 162 cases were identified as *T. solium* distributed across 10 provinces, notably in the north-eastern part of the country, while 1399 cases were identified as *T. saginata*, distributed across four provinces, mostly in the western part of the country [75].

Over the past 20 years, the Office for Controlling and Eliminating Taeniasis and Cysticercosis was gradually scaled back due to the strong decline of cysticercosis in many areas [72]. At present, some pilots are being carried out, one in Henan province in the central part of the People’s Republic of China, where cysticercosis was once highly endemic [76]. Another two control pilots were set up in the provinces of Yunnan and Sichuan in the south-western part of the country with the aim of supporting cysticercosis control through IEC, WASH, screening cases with suspected taeniasis, and providing treatment when needed.

### Imported NTDs

The recent, substantial increase in population mobility, including long-haul travels, led to importation of various NTDs into the People’s Republic of China [77]. For instance, imported cases of human African trypanosomiasis and schistosomiasis haematobia have been reported. Fortunately, these diseases do not transmit within the People’s Republic of China, owing to the absence of vectors (tsetse fly for human African trypanosomiasis) and intermediate hosts (snails of the genus *Bulinus* for schistosomiasis haematobia) [78, 79]. Although no indigenous transmission of schistosomiasis mansoni has been reported thus far, attentions should be paid to the importation and current presence of the *S. mansoni* intermediate host *Biomphalaria straminea* in the southern part of the People’s Republic of China [78, 80].

Importation of visceral leishmaniasis cases worsens further control and planned elimination of this disease in the People’s Republic of China. In addition, epidemics of dengue in neighbouring Southeast Asia constitute a high risk for this disease also in the People’s Republic of China, as transmission of diseases does not respect national borders, as shown by an outbreak in the southern parts of the country in 2014 [81]. Additionally, the importation of freshwater shellfish products might cause outbreaks of food-borne trematode infections [82].

## Drives for NTDs control and elimination in the People’s republic of China

### Social and economic development

**Fig. 3:**
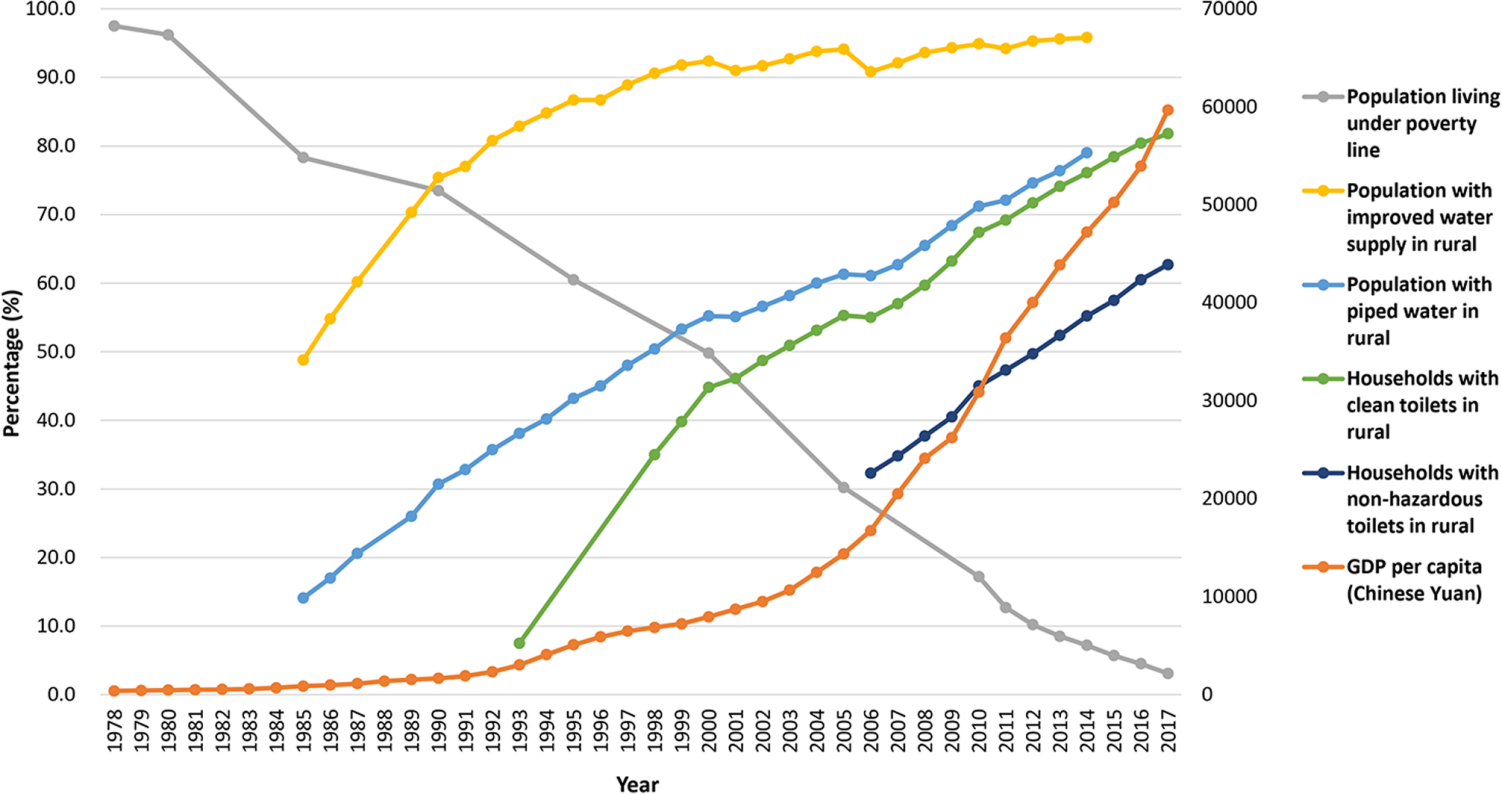
Change trends of GDP per capita, sanitation, water supply, and poverty reduction in the People’s Republic of China. GDP: Gross domestic product

Since the 1950s, the control of schistosomiasis, leishmaniasis, hookworm infection, and lymphatic filariasis were given high priority in the plans for national development, while attention was also paid to leprosy and trachoma [24]. Still today, the integrated public health activities of the Patriotic Public Health Programme play important roles in the control of many infections [31]. This approach was initiated in the early 1950s, focussing on improved sanitation and construction of water works, while it is also tasked with general vector control (flies, mosquitoes, cockroaches, and mice). For example, people living in rural areas of the People’s Republic of China benefitted from improved access to clean water, which increased from 48.8% in 1985 to 95.8% in 2014, while access to piped water has increased from 14.1 to 79.0% (Fig. 3) [83–86]. Although the percentage of households with latrines in rural parts of the People’s Republic of China reached 85.9% in 1993, the percentage with clean latrines was only 7.5% which, however, increased to 81.8% in 2017 [86, 87]. The coverage of non-hazardous toilets (with pathogens killed) increased from 32.3% in 2006 to 62.7% in 2017 [83, 86].

Economic development is a key driver for the control and elimination of NTDs. The per capita gross domestic product (GDP) has increased from 385 Chinese Yuan in 1978 to as much as 59 660 Chinese Yuan in 2017 (Fig. 3) [88]. Nearly all NTDs are associated with poverty and are therefore more of a problem in the least developed areas. The population defined as living under the poverty line in the People’s Republic of China has decreased from 770.4 million in 1978 to 30.5 million in 2017, which means that the rate of poverty decreased from 97.5 to 3.1% [88].

### Epidemiological surveys and surveillance

Understanding the epidemiology, including the spatial and temporal risk of NTDs, is the first step before implementing control and elimination efforts. Several large-scale epidemiological surveys have been carried out, and for several NTDs, the surveys are regularly repeated to monitor progress and identify areas that need special attention. There is considerable granularity across NTDs. For example, while county-level risk maps have been available for leishmaniasis and lymphatic filariasis for several decades [13, 37], the risk map for schistosomiasis is constantly updated, and thus contains accurate village-level risk profiles [64, 66]. The national survey in 2012–2016 pertaining to echinococcosis produced a county-level map [44], while the three national surveys on intestinal helminthiases have been updated to provide information at the sub-provincial level for soil-transmitted helminth infection, clonorchiasis, and taeniasis [42, 51, 56].

These epidemiological surveys take into account the large population of the People’s Republic of China, enrolled hundreds of thousands of participants, and are indeed the largest in the world. For instance, more than one million people were included in the national survey pertaining to echinococcosis in 2012–2016 and more than 600 000 participants were enrolled in the third national survey for intestinal helminthiases in 2014–2015. Taken together, the People’s Republic of China has established a large surveillance system for infectious diseases, which includes both an active and a passive system (Table 1) [20]. Nowadays, active population surveys cover all endemic villages for schistosomiasis, all endemic provinces for echinococcosis, all indigenous endemic provinces for dengue, several major endemic provinces for rabies, and nearly all provinces where soil-transmitted helminth infection and clonorchiasis occur. The passive surveillance system based on reports from national medical organisations covers all provinces, in which seven NTDs are included, namely leprosy, dengue, rabies, leishmaniasis, echinococcosis, lymphatic filariasis, and schistosomiasis [20].

### Research and development

National, provincial, prefectural, and county-level anti-infectious stations (nowadays as the Centers for Disease Control and Prevention), have been established throughout the People’s Republic of China [31]. In addition to these four levels, many special anti-infectious organisations were set up in the endemic areas; specifically those targeting schistosomiasis, lymphatic filariasis, leishmaniasis, and leprosy. These organisations play important roles in innovating and rigorously validating new diagnostics, drugs, and vaccines, as well as designing disease control strategies, and conducting operational and implementation research.

Particular attention is paid on the development of novel diagnostic techniques that are adapted to the specific stage of a disease control programme. Usually, besides diagnosis of the pathogen, the behavioural screening, serological and molecular techniques are applied at different stages [89, 90]. For example, behavioural screening should be applied at the morbidity control stage, owing to the simplicity and low cost, while molecular techniques are warranted in the pre-elimination stage, while serology is particularly useful in the late stages of infection and transmission control. In line with the global NTD control strategy put forth by WHO emphasising preventive chemotherapy, drugs and drug development are also highly prioritised, including research on new formulations and exploring novel implementation strategies. Tribendimidine, developed by Chinese scientists, has been approved for the treatment of soil-transmitted helminth infection in the People’s Republic of China [91]. Recently, clinical trials also showed high efficacy of tribendimidine against *C. sinensis* [92, 93]. In order to increase coverage and compliance, new formulations have been developed. Two prominent examples are the distribution of diethylcarbamazine-fortified salt against lymphatic filariasis, and the addition of sugar to anthelminthics used against soil-transmitted helminth infections [13, 60, 94].

In addition to integrated control activities, namely the Patriotic Public Health Programme [31], a special strategy is usually explored for each NTD. Although several NTDs are co-endemic in specific provinces, the overlap might not be too extensive, depending on the social-ecological contexts. A strategy is usually established based on the complete understanding of determinants, and the available techniques, human and financial resources [63]. Pilot studies, coupled with operational research and cost-effectiveness considerations, are regularly implemented [60, 95]. Strategies that proved successful drive large-scale implementation. Different strategies are usually adopted for specific endemic situations and they are adjusted and fine-tuned over time, based on surveillance data.

## Targets beyond 2020

### Targets

Elimination of lymphatic filariasis as a public health problem has been achieved in 2007 (Table 2) [13]. Trachoma was announced to be eliminated as a public health problem in 2015 [14]. On one hand, this achievement needs to be further strengthened, while on the other hand, its elimination needs to be verified by WHO.

**Table 2:** Control and elimination targets of NTDs in the People’s Republic of China and at the global level

Infection	Global goal by 2020	Situation in the People’s Republic of China		
		Current stage	2020 goals	Strategy
Bacterial				
Leprosy	Global elimination	Transmission control	Case no. decreased by 50% compared to 2010; Prevalence decreased to less than 1/100 000 in almost all counties (98%); Type 2 disability < 20% in new cases	Integrated strategy through early detection and regular treatment, surveillance, information, education, and communication (IEC) and prevention of disability
Trachoma	Global elimination	Elimination as a public health problem in 2015	–	Surveillance, information, education, and communication (IEC)
Viral				
Dengue	Control and surveillance systems in all regions; Case number reduced by > 25% and deaths by 50% compared to 2009–2010 baseline	Infection control	No	Surveillance; vector control
Rabies	Regional elimination in Southeast Asia and Western Pacific in 2020	Transmission control	Achievement of the national control (the indicators in human beings is unclear)	Multiple measures including surveillance and response; dog immunization (> 90% of registered dogs) and strengthening of diagnostic capacity
Protozoal				
Leishmaniasis	Regional elimination of visceral leishmaniasis in Indian subcontinent	Transmission control	Burden strongly decreased	Provision of long-lasting insecticidal nets, vector elimination and the control of transmission sources in areas with dogs, staff training for early detection, treatment, and surveillance
Helminth				
Echinococcosis	Validated strategy available and interventions scaled up in selected countries	Morbidity control	The prevalence decreased to < 1% in humans and < 5% in domestic dogs in > 70% endemic counties	Integrated strategy including control of transmission sources and management of intermediate hosts, detection and treatment of human cases
Food-borne trematodiasis	Preventive chemotherapy achieved for 75% of all populations at risk; morbidity controlled in all endemic countries	Morbidity control	Prevalence decreased by 30% in major endemic areas compared to that in 2015	Integrated strategy, including information, education, and communication (IEC) and control of transmission sources (such measures as water supply and improvement of sanitation, environmental changes, behaviour change and chemotherapy)
Lymphatic filariasis	Global elimination	Eliminated as public health problem in 2007	–	Surveillance
Schistosomiasis	Regional elimination in Southeast Asia and selected countries in Africa	Transmission control	National elimination by 2030	Integrated strategy, especially control of transmission source
Soil-transmitted helminth infection	75% of pre-school and school-aged children in need of treatment regularly treated; 75% coverage achieved in pre-school and school-aged children in all countries	Infection control	The prevalence decreases by 20% in major endemic areas compared to that in 2015	Integrated strategy including information, education, and communication (IEC) and control of transmission sources (such measures as water supply and improvement of sanitation, environmental changes, behaviour change, and chemotherapy)
*Taenia solium* taeniasis and cysticercosis	Interventions scaled up in selected countries for control and elimination	Infection control	Validated control strategy for high endemic areas and elimination strategy for low endemic areas	Integrated strategy including information, education, and communication (IEC), detection and treatment of cases, improvement of sanitation and provision of water in highly endemic areas and establishment of surveillance in other areas

*NTDs* Neglected tropical diseases

Leprosy, schistosomiasis, leishmaniasis, and rabies are currently all in the lowest-endemic level, namely a stage characterised by transmission control (Table 2). It is already announced that schistosomiasis will be at the stage of elimination by 2030 [68]. In view of achievements made thus far, this target seems eminently reasonable. The progress towards the elimination of leprosy will be further strengthened. Over 98% counties are expected to have a prevalence < 1/100 000 by 2020 [22]. Although the targets of leishmaniasis and rabies have yet to be defined, these two NTDs are targeted for elimination in the near future.

The soil-transmitted helminths, *T. solium* taeniasis/cysticercosis, and dengue are at the stage of infection control (Table 2). This means that by 2020 the prevalence of the soil-transmitted helminth infection in high endemic provinces should decrease by 20% compared to that estimated in 2015 [41]. Elimination pilots have been undertaken for *T. solium* taeniasis and cysticercosis [76].

Compared to other NTDs, echinococcosis and the food-borne trematode infections are still at the stage of morbidity control constituting important public health problems in the western and eastern parts of the People’s Republic of China, respectively (Table 2). The national goal is to decrease the prevalence of echinococcosis to less than 1% in humans and less than 5% in domestic dogs in over 70% of the endemic counties by 2020. In addition, the prevalence of food-borne trematode infection should be reduced with 30% in major endemic areas by 2020, as compared to 2015 [41].

### Challenges and opportunities

Although considerable progress has been achieved in the control and elimination of NTDs since the founding of the People’s Republic of China exactly 70 years ago, challenges remain at the onset of the Belt and Road Initiative [96]. Due to the permissible environment and lower level of economic development in the western parts of the country, many NTDs remain endemic there and infrastructure is still underdeveloped (Table 1 and Fig. 1). The zoonotic nature of some of the NTDs, such as rabies, echinococcosis, clonorchiasis, and taeniasis/cysticercosis, holds a permanent risk of emergence. There are deeply rooted habits of raw food consumption, such as raw pork in western and raw freshwater fish in the eastern parts of the country [46, 76]. Economic development usually is associated with declines of the risk of NTDs. However, some NTD outbreaks have been linked to people trying new dietary habits that emerged due to enhanced socio-economic status. Urbanization and concentration of people in big cities increase the risk for outbreaks of vector-borne NTDs, especially dengue [97]. Besides the importation of NTDs from other part of the world, huge population movements within the country confront control and elimination efforts of many NTDs. Due to the imbalance of economic development, large numbers of people move from less developed areas to the big cities, which entails the transfer of some NTDs from the western to the eastern parts of the People’s Republic of China (e.g. schistosomiasis and leishmaniasis) [39, 66] and when people visit their home villages, other NTDs might come with them (e.g. dengue) [28]. Additionally, climate change should be taken seriously as it might redraw the epidemiological maps over the coming decades. Already, some vectors (e.g. mosquitoes transmitting dengue) and the intermediate host snails for schistosomiasis have started to do so [98, 99].

In 2016, the government of the People’s Republic of China launched a plan to eliminate poverty by 2020, which will contribute to the control and elimination of NTDs, particularly in the least developed areas [100]. A national plan of action, known as “Healthy China 2030” has been established with health being integrated into each policy enacted [101]. WASH will be further improved. Specifically, the water supply in rural areas will be upgraded and the coverage of non-hazardous toilets will be increased. “Toilet revolution” is a slogan that gained traction in recent times [102]. This approach is planned to result in the coverage of clean toilets exceeding 85% by 2020 in rural areas, leading to a complete coverage of non-hazardous toilets for the country as a whole by 2030 [103].

## Conclusions

Since the founding of the People’s Republic of China in 1949, considerable progress has been made in the control and elimination of NTDs. This not only benefits the local communities with respect to health and wellbeing, but also promotes progress towards ending the global presence of the NTDs. Furthermore, experiences and lessons in controlling and eliminating NTDs in the People’s Republic of China have attracted worldwide recognition, and hence, innovations and control strategies are stimulating other parts of the world, especially Africa, Latin America, and Southeast Asia where NTDs remain a public health problem. The inclusion of NTDs in the Belt and Road Initiative will not only promote the global control of NTDs but also consolidate the achievements of this initiative.

## Supplementary information

**Additional file 1:** Multilingual abstracts in the five official working languages of the United Nations.

## Data Availability

All data supporting the findings of this study are included in the article.

## Abbreviations

DALYs: Disability-adjusted life years
IEC: Information, education, and communication
NTDs: Neglected tropical diseases
WASH: Water, sanitation, and hygiene
WHO: World Health Organization
