# Supplementary material for: Neglected tropical diseases in the People’s Republic of China: progress towards elimination
Source: Infect Dis Poverty. 2019 Oct 2;8:86. doi: 10.1186/s40249-019-0599-4 (PMC6775666; doi:10.1186/s40249-019-0599-4)
Supplement: Supplementary file 1 — Additional file 1: Multilingual abstracts in the five official working languages of the United Nations. [file 40249_2019_599_MOESM1_ESM.pdf]

Translation of the abstract into the five official working languages of the United Nations

**الأمراض الإستوائية المهملة في جمهورية الصين الشعبية: التقدم نحو القضاء النهائي**

مين باو تشيان ، جين تشن ، روبرت بيرجكويست ، تشونغ جي لي ، شي تشو لي ، نينغ شياو ، يورغ أوتزينجر ، شياو نونغ تشو

**الملخص**

منذ تأسيس جمهورية الصين الشعبية في عام ١٩٤٩ تم إحراز تقدما كبيرا في المقاومة والقضاء على المجموعة الأولية التي تمثل إحدي عشر مرضا من أمراض المناطق الإستوائية المهملة وبالفعل تم الإعلان عن التخلص من مشكلة داء الفيلاريات اللمفاوي كمشكلة صحية عامة في عام ٢٠٠٧ والتراخوما في عام ٢٠١٥ وكانت الأعداد الباقية من الأشخاص المصابين بعدوى الطفيليات المنقولة عن طريق التربة وداء متفرعات الخصية وداء الشريطيات وداء المشوكات في عام ٢٠١٥ تبلغ ٢٩,١ مليون و ٦,٠ مليون و ٣٦٦٢٠٠ و ١٦٦١٠٠ على التوالي وفي عام ٢٠١٧ وذلك بعد أكثر من ٦٠ عامًا من المكافحة متعددة الجوانب والغير منقطعة للبلهارسيا شهد عدد الحالات المصابة بالمرض انخفاضًا من أكثر من ١٠ ملايين إلى ٣٧٦٠٠ حالة وفي الوقت نفسه تم الإبلاغ عن حوالي ٦٠٠٠ حالة حمى الضنك في حين إنخفضت حالات الإصابة بداء الليشمانيات والجذام وداء الكلب إلي ٦٠٠ حالة أو أقل في السنة ويعد توفير التنمية الاجتماعية والاقتصادية المستدامة جنبًا إلى جنب مع تحسين المياه والصرف الصحي والنظافة الصحية أساسًا للتقدم المستمر في حين أن المراقبة الصارمة واستجابات الصحة العامة المحددة ستدعم الإنجازات وتحدد جدول زمنيا للقضاء النهائي علي هذه الأمراض ولهذا فإن أهداف القضاء على الفقر والخطط الاستراتيجية وحزم التدخل بعد عام ٢٠٢٠ تمثل فرصًا مهمة لمزيد من المقاومة والقضاء النهائي عندما تتطلب التحديات المتبقية بذل جهود مستدامة.

Translated from English version into Arabic by Mohamed R. Habib

**中国的被忽视热带病：迈向消除**

Men-Bao Qian, Jin Chen, Robert Bergquist, Zhong-Jie Li, Shi-Zhu Li, Ning Xiao, Jürg Utzinger, Xiao-Nong Zhou

**摘要**

自中华人民共和国 1949 年成立以来，曾严重流行的 11 种被忽视热带病在控制和消除方面取得了显著的成就。2007 年实现淋巴丝虫病消除；2015 年实现沙眼消除；2015 年土源性蠕虫病、华支睾吸虫病、带绦虫病和棘球蚴病的患病人数分别为 2910 万、600 万、366200 和 166100。经过 60 余年不懈的努力，血吸虫病患病人数已由建国初期的 1000 多万降到 2017 年的 37600。2017 年，登革热报告病例数为 6000 余例，而利什曼病、麻风病和狂犬病新发病例数每年均在 600 例左右或以下。社会和经济的持续发展以及饮水、厕所和个人卫生的不断改善推动了防治进程；监测和专项的公共卫生防治工作巩固了防治成就并且促进向消除的方向迈进。2020 年消除贫困以及各项高屋建瓴的策略和防治规划为中国进一步控制乃至消除被忽视热带病提供了契机，但仍存在一些挑战需要我们持续不断地努力。

Translated from English version into Chinese by Men-Bao Qian

**Maladies tropicales négligées en République Populaire de Chine: Progrès vers l'élimination**

Men-Bao Qian, Jin Chen, Robert Bergquist, Zhong-Jie Li, Shi-Zhu Li, Ning Xiao, Jürg Utzinger, Xiao-Nong Zhou

**Résumé**

Depuis la création de la République Populaire de Chine en 1949, des progrès considérables ont été réalisés dans la lute et l'élimination de l'ensemble initial de 11 maladies tropicales négligées en Chine. En effet, l'élimination en tant que problème de santé publique a été déclarée pour la filariose lymphatique en 2007 et pour le trachome en 2015. Le nombre restant de personnes touchées par les vers parasitaires

transmis par le sol, la clonorchose, la téniose et l'échinococcose en 2015 était de 29,1 millions, 6 millions, 366 200 et 166 100, respectivement. En 2017, après plus de 60 ans de lutte intégrée contre la schistosomiase, le nombre de cas est passé de plus de 10 millions à 37 600. Entre-temps, environ 6 000 cas de dengue sont signalés, tandis que l'incidence de la leishmaniose, de la lèpre et de la rage est en baisse à 600 ou moins par an. Un développement social et économique soutenu, allant de pair avec l'amélioration de l'accès à l'eau potable, de l'assainissement et de l'hygiène, constitue la base de progrès continus, tandis qu'une surveillance rigoureuse et des mesures spécifiques de santé publique permettront de consolider les acquis et de définir le programme d'élimination. Les objectifs d'élimination de la pauvreté, les plans stratégiques et les programmes d'intervention après 2020 sont d'importantes occasions de poursuivre le contrôle et l'élimination de la pauvreté, lorsque les défis qui restent à relever exigent des efforts durables.

Translated from English version to French by Golou Louise Bellai, and corrected by Jürg Utzinge

**Заброшенные тропические болезни в Китайской Народной Республике: прогресс на пути к ликвидации**

Men-Bao Qian, Jin Chen, Robert Bergquist, Zhong-Jie Li, Shi-Zhu Li, Ning Xiao, Jürg Utzinger, Xiao-Nong Zhou

**Аннотация**

Со времени основания Китайской Народной Республики в 1949 году был достигнут значительный прогресс в борьбе и ликвидации первоначального набора в 11 странах заброшенных тропических болезней. Действительно, элиминация как проблема общественного здравоохранения была объявлена для лимфатического филяриатоза в 2007 году и для трахомы в 2015 году. Оставшееся число людей, затронутых почвенной инфекцией гельминтов, клонорхозом, таениозом и эхинококкозом в 2015 году, составило 29,1 миллиона, 6,0 миллиона, 366 200 и 166 100 соответственно. В 2017 году, после более чем 60 лет непрерывного многопланового контроля над шистосомозом, число случаев сократилось с более чем 10 миллионов до 37 600 человек. Между тем, сообщается о 6000 случаях заболевания денге, в то время как заболеваемость лейшманиозом, проказой и бешенством снижается до 600 или менее в год. Устойчивое социально-экономическое развитие, идущее рука об руку с улучшением водоснабжения, санитарии и гигиены, обеспечивает основу для дальнейшего прогресса, в то время как строгий надзор и конкретные ответные меры общественного здравоохранения консолидируют достижения и формируют повестку дня ликвидации. Цели по искоренению бедности, а также стратегические планы и пакеты мер на период после 2020 года являются важными возможностями для дальнейшего контроля и ликвидации, когда сохраняющиеся проблемы требуют устойчивых усилий.

Translated from English version into Russian by Hao-Qi Zhang

**Enfermedades tropicales desatendidas en la República Popular de China: progreso hacia la eliminación**

Men-Bao Qian, Jin Chen, Robert Bergquist, Zhong-Jie Li, Shi-Zhu Li, Ning Xiao, Jürg Utzinger, Xiao-Nong Zhou

**Resumen**

Desde la fundación de la República Popular China en 1949, se ha logrado un considerable progreso en el control y la eliminación de aquellas 11 enfermedades tropicales desatendidas, inicialmente seleccionadas por el país. De hecho, la eliminación de la filariasis como problema de salud pública fue declarada en 2007 y del tracoma en 2015. El número de personas aún afectadas por geohelminthiasis,

clonorquiasis, teniasis y equinococosis en 2015 fue de 29,1 millones, 6,0 millones, 366 200 y 166 100, respectivamente. En el 2017, después de más de 60 años de control ininterrumpido de la schistosomiasi mediante múltiples intervenciones, el número de casos disminuyó de más de 10 millones a 37 600. Al mismo tiempo, se informan de unos 6 000 casos de dengue, mientras que la incidencia de leishmaniasis, lepra y rabia se redujo a 600 o menos por año. El desarrollo social y económico sostenido, acompañado de mejoras en el agua, el saneamiento y la higiene, proporciona la base para un progreso continuo, mientras que la vigilancia rigurosa y las respuestas específicas de salud pública consolidarán los logros y darán forma a la agenda de eliminación. Las metas de eliminación de la pobreza, los planes estratégicos y los paquetes de intervención posteriores a 2020 son importantes oportunidades para impulsar el control y la eliminación, cuando los desafíos pendientes requieran de esfuerzos sostenibles.

Translated from English to Spanish by Anja Schreier & Louise Segura
